# Challenges for the Self-Assembly of Poly(Ethylene Glycol)–Poly(Lactic Acid) (PEG-PLA) into Polymersomes: Beyond the Theoretical Paradigms

**DOI:** 10.3390/nano8060373

**Published:** 2018-05-26

**Authors:** Alexsandra Conceição Apolinário, Monika S. Magoń, Adalberto Pessoa Jr., Carlota de Oliveira Rangel-Yagui

**Affiliations:** 1 Department of Biochemical and Pharmaceutical Technology, School of Pharmaceutical Sciences, University of São Paulo, Av. Prof. Lineu Prestes, 580-Bl.16, São Paulo 05508-000, Brazil; acapolinario@gmail.com (A.C.A.); pessoajr@usp.br (A.P.J.); 2 Department of Chemistry, University College London, Christopher Ingold Building, 20 Gordon Street, London WC1H 0AJ, UK; m.magon.12@ucl.ac.uk; 3 Current address: BSRC Complex, School of Biology, University of St Andrews, St Andrews KY16 9ST, UK

**Keywords:** amphiphilic block copolymers, polymeric vesicles, self-aggregated nanostructures, post-film hydration steps

## Abstract

Polymersomes (PL), vesicles formed by self-assembly of amphiphilic block copolymers, have been described as promising nanosystems for drug delivery, especially of biomolecules. The film hydration method (FH) is widely used for PL preparation, however, it often requires long hydration times and commonly results in broad size distribution. In this work, we describe the challenges of the self-assembly of poly (ethylene glycol)-poly(lactic acid) (PEG-PLA) into PL by FH exploring different hydrophilic volume fraction (*f*) values of this copolymer, stirring times, temperatures and post-FH steps in an attempt to reduce broad size distribution of the nanostructures. We demonstrate that, alongside *f* value, the methods employed for hydration and post-film steps influence the PEG-PLA self-assembly into PL. With initial FH, we found high PDI values (>0.4). However, post-hydration centrifugation significantly reduced PDI to 0.280. Moreover, extrusion at higher concentrations resulted in further improvement of the monodispersity of the samples and narrow size distribution. For PL prepared at concentration of 0.1% (*m*/*v*), extrusion resulted in the narrower size distributions corresponding to PDI values of 0.345, 0.144 and 0.081 for PEG_45_-PLA_69_, PEG_114_-PLA_153_ and PEG_114_-PLA_180_, respectively. Additionally, we demonstrated that copolymers with smaller *f* resulted in larger PL and, therefore, higher encapsulation efficiency (EE%) for proteins, since larger vesicles enclose larger aqueous volumes.

## 1. Introduction

In the last few years, polymersomes (PL), vesicles composed of amphiphilic-block copolymers, have attracted attention in the pharmaceutical field as versatile nanostructures with colloidal stability, tunable membrane properties and ability to encapsulate a broad range of hydrophilic and hydrophobic drugs including biomacromolecules with therapeutic potential, such as proteins [1]. The use of nanotechnology to deliver biological drugs such as monoclonal antibodies, antibody fragments, peptides, replacement factors, enzymes and vaccines is increasing exponentially. Encapsulation of these active compounds into PL may provide superior benefits in terms of decreasing immunogenicity, increasing biomolecule’s half-life and stabilizing these mostly protein-based drugs against denaturation and enzymatic degradation [2]. The spontaneous and reversible organization of amphiphilic molecules of block copolymers into supramolecular structures such as PL is known as self-assembly and it is certainly one of the most promising technologies in pharmaceutical nanoscience, yet it still requires in-depth studies in order to be fully understood and employed in therapeutics strategies [3]. 

In principle, PL formation could be achieved through simple contact of amphiphilic copolymers with water due to the hydrophobic effect [4]. Nevertheless, despite the spontaneity of self-assembly, reports have shown that a variety of energy-intensive protocols, such as stirring, sonication and extrusion, are necessary to obtain uniform PL samples [5,6]. Furthermore, the reversible organization is counteracted by the nature of block copolymers with the critical aggregation concentration (CAC.) near to zero, and negligible exchange of material within PL or with the surrounding solution [7].

The copolymer nature dictates different properties of PL systems suggesting that methods for self-assembly of copolymers into PL could be a challenging process that entails different platforms depending on the copolymer used. The main theory for self-assembly of copolymers into PL reported by the pioneer researches in the field is based on the value of hydrophilic volume fraction (*f*) that predicts if an amphiphilic molecule aggregates into lamellae, micelles or vesicles in aqueous solutions at equilibrium concentration [8]. However, this parameter does not assure the copolymers self-assembly into PL; the preparation conditions are also a key factor [9]. One of the most employed methods to prepare self-aggregated nanostructures is the film hydration (FH). However, FH may lead to broad and multimodal PL size distributions [10] and usually complimentary steps are required to obtain vesicles with narrow size distribution. Among these complimentary steps, one can employ the extrusion of the dispersion through a polycarbonate membrane with a certain pore size or size exclusion chromatography (SEC.) [11].

In this work, we investigated the challenges of poly (ethylene glycol)–poly (lactic acid) (PEG-PLA) self-assembly into PL. All *f* values of copolymers were in the theoretical range reported to obtain vesicles (25–40%), but we used PEG-PLA with low and high molecular weight (MW) and different *f* values to see the influence of this parameter on vesicle formation and protein encapsulation capacity: PEG_45_-PLA_69_ PEG_114_-PLA_153_ and PEG_114_-PLA_180_. The method employed, film hydration, is a bottom-up approach and different stirring times, temperatures and copolymer concentrations were studied. Finally, we also employed different post-hydration steps in an attempt to reduce PL broad size distributions. 

## 2. Materials and Methods 

The poly (ethylene glycol)–poly(lactic acid) (PEG-PLA) amphiphilic block copolymers were obtained from Polymer Source^®^ (Montreal, QC, Canada). Phosphotungstic acid, bovine serum albumin (BSA) and the bicinchoninic acid (BCA) were purchased from Sigma Aldrich (Sigma Aldrich Co., Saint Louis, MO, USA)**.** l-asparaginase (225 U/mg) was acquired from ProSpec Tany^®^ (Ness-Ziona, Israel). All other reagents were of analytical grade and purchased from Synth^®^ (São Paulo, São Paulo, Brazil) and water ultra-purified in a Milli-Q system (Merck Millipore, Billerica, MA, USA) was used in all experiments.

### 2.1. Selection of Amphiphilic Copolymer

The selection of the copolymers was based on the differences between the hydrophilic (PEG) and hydrophobic (PLA) molecular masses; copolymers with hydrophilic fraction (*f*) between 0.20 and 0.42 and of different total molecular weight were used. Table 1 shows the molecular characteristics of the three PEG-PLA copolymers chosen.

### 2.2. Film Hydration

The polymeric film obtained was hydrated with PBS 1X pH 7.4 resulting in a system with PEG-PLA at 0.03 % (*m*/*v*). For film hydration, we employed orbital agitation in a rotary-evaporator Büchi^®^ R-210 (Flawil, Switzerland) at 150 RPM by 2 h or overnight, or magnetic stirring in a Corning PC-420D^®^ agitator (Chelmsford, UK) at 400 RPM by 12, 24, 48 and 72 h, at room temperature or 40 °C. In an attempt to narrow size distribution, sonication was performed in a Qsonica^®^ ultrasound bath (Columbiana, OH, USA) at 50W (by 20 or 50 min).

### 2.3. Centrifugation and Extrusion

Systems obtained after film hydration were centrifuged at 2000× *g* for 5 min in an Eppendorf 5810 R^®^ centrifuge (Eppendorf, Hamburg, Germany). The supernatant was considered as purified sample. Extrusion was performed passing the systems 31 times through a 400 nm pore radii polycarbonate membrane in an Avanti Polar Lipids^®^ extruder (Avanti Polar Lipids, Inc., Alabaster, AL, USA), at 40 °C.

### 2.4. Dynamic Light Scattering (DLS)

DLS analysis was performed in a Zetasizer Nano ZS (Malvern Instruments, Worcestershire, UK) with a 633 nm HeNe laser at 90°. The samples were analyzed as obtained (without filtration) to ensure that large populations were not rejected and, therefore, the challenges of hydration film method in each condition tested could be observed. Polydispersity indices (PDI) were obtained from the correlation function by cumulant analysis using the Malvern software (producer, city, state, country), which was then analyzed by non-negative least squares to obtain the intensity-weighted distribution of diffusion coefficients (*D_f_*). The autocorrelation functions in DLS is calculated by data fitting and then the *D_f_* is calculated using Equation (1). The hydrodynamic radius (R_h_) of solid spherical particles can be derived as shown in Equation (1) (Stokes-Einstein equation).

(1) $$D_{f}=\frac{k_{B}\;T}{6{\pi \eta R}_{h}}$$

in which: k*_B_* = Boltzmann constant (1.38064852 × 10^−23^ J/K), *T* = temperature, η = absolute viscosity and R_h_ = hydrodynamic radius.

The average R_h_ of the polymersomes (and consequently the hydrodynamic diameter, D_h_) was determined from intensity weighted and number-weighted distributions, assuming non-interacting particles modeled as homogeneous hard spheres. Each solution was analyzed at least 3 times depending on the observed correlogram. Whereas this assumption might be suitable for spherical vesicles, it is not very precise for mixed systems with tubular vesicles or aggregates with varied shapes. Nonetheless it might give good indications and facilitate comparison of the different samples. 

### 2.5. Nanoparticle Tracking Analysis (NTA) 

NTA was performed with a Nanosight^®^ LM14 instrument (Malvern Instruments, Worcestershire, UK). In this technique, samples are illuminated by a focused (80 µm) beam of a single mode laser diode (405 nm) and the light scattered by the particles in then tracked.

### 2.6. Transmission Electron Microscopy 

The morphology of aggregates was observed by transmission electron microscopy (TEM) in a Tecnai G20 microscope (FEI Company, Hillsboro, OR, USA), with accelerating voltage of 80 kV. For the analysis, 5μL of each system was placed on a copper grid and coated with a thin carbon film; phosphotungstic acid (1.0 %) was used as negative staining. 

### 2.7. Encapsulation of Model Globular Protein BSA and Therapeutic Protein l-Asparaginase

To further prove that vesicles were formed and to check their capacity for protein encapsulation, bovine serum albumin (BSA, 66 KDa) and the anti-leukemic enzyme l-asparaginase (ASNase, 142 kDa) were encapsulated in the polymersomes. For this purpose, we employed the best conditions for polymersomes preparation (film hydration at room temperature, with magnetic stirring at 400 RPM by 24 h and extrusion by 400 nm pore membranes at 40 °C) replacing the PBS for a 1 mg/mL BSA or ASNase solution for film hydration. The encapsulation efficiency (EE%) was determined by indirect method (Equation (2)) after centrifugation at 10,000 × *g* for 30 min. The concentration of unencapsulated protein on the supernatant was determined by the bicinchoninic acid (BCA) method following the manufacture’s protocol (Sigma Aldrich, St Louis, MO, USA).

(2) $$EE_{\%}=\left(\frac{{\;P}_{\mathrm{total}\;}-{\;P}_{\sup}}{P_{\mathrm{total}\;}\;}\right)\;\times \;100\;$$

in which: P_total_ is the total protein mass added into the system, P_sup_ is is the protein mass in the supernatant after centrifugation.

### 2.8. Statistical Analysis

Data was treated in the Origin Pro 8^®^ software (Originlab Corporation, Wellesley, MA, USA). Student's t-test or one-way analysis of variance (ANOVA) and Tukey post hoc were used to evaluate the PDI values found for the systems obtained from different copolymers under different conditions.

## 3. Results and Discussion

### 3.1. Self-Assembly by Film Hydration under Orbital Agitation Versus Magnetic Stirring

First assays were performed with PEG_45_PLA_69_ (2000:5000 KDa) before testing other copolymers. The results indicated that film hydration by agitation in a rotary-evaporator resulted in the presence of large polymeric bulk material (Figure S1A) and bimodal distribution with high polydispersity index (PDI) values (Table 2) even after sonication. The orbital process does not exert a direct shear force on the hydration liquid and, therefore, leads to detachment of large slices of the film from the surface of the glass flask. This bulky polymer film may remain in the solution and coexist with vesicles and/or micelles, resulting in large polydispersity and/or bimodal size distribution [11,12]. Although literature reports that bulk polymeric film may coexist with PL and other aggregates [13], these bulk polymeric films sediment out of the solution in dilute conditions in which intermolecular interactions between the vesicles have no significant role in maintaining the bulk film dispersed in solution. Those systems may be interesting only for individual assembly characterization and are not adequate for drug encapsulation due to heterogeneity and potential loss of the material upon sedimentation [14].

To avoid the presence of bulky polymer after film hydration, magnetic stirring overnight was employed. The macroscopic difference in the system was remarkable and sedimentation was not observed (Figure S1B). Magnetic stirring improved the copolymers self-assembly since the shear force leads to detachment of the unillamelar polymeric film from the flask surface and favors film breaking into smaller vesicular structures [15]. Diffusion of the aqueous phase occurs on a larger surface area, which in turn favors the hydrophobic effect and polymeric vesicle formation. In contrast, orbital agitation with lower shear forces allowed only larger bulk polymeric film pieces to detach, which leads to limited aqueous phase penetration into the film. TEM was not performed for these systems since broad size distributions were observed. Nonetheless, either PL or other complex self-assembled nanostructures were formed.

Following, we investigated the influence of polymer MW and hydrophilic fraction (*f*) on PL polydispersity. Table S1 shows the results for size distribution by intensity and number based on dynamic light scattering (DLS) measurements after overnight hydration by magnetic stirring. As already mentioned, wide size distributions are inherent to the self-assembly since this technique is not precise in producing nanostructures with a good control of size [8]. One of the reasons for this is that the energetic penalty involved in amphiphiles self-assembly into vesicles from a membrane with zero natural net curvature results from the sum of the mean and Gaussian curvature and not from the radius [5].

**Figure 1:**
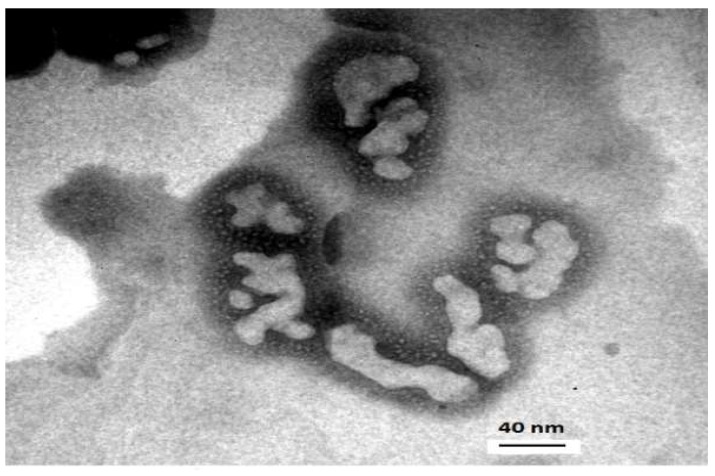
Transmission electronic microscopy of polymeric non-spherical aggregates formed from poly (ethylene-block-lactic acid) 5000:11,000 after overnight magnetic stirring.

Even with lower visual polymeric bulk film sedimentation, the DLS indicated a broad distribution of size with high PDI values. Cumulants fit were not used to analyze the z-average hydrodynamic radius, instead the scattering profile by number distribution was presented in Table S1 in order to avoid over representation of large objects that we believe are part of smaller populations of remaining non-spherical and irregular aggregates (Figure 1). The presence of these large structures even in lesser proportions causes significant light scattering and occupy a larger volume in the sample as compared to the smaller structures that might constitute the major population by number distribution in the heterogenous system. 

**Figure 2:**
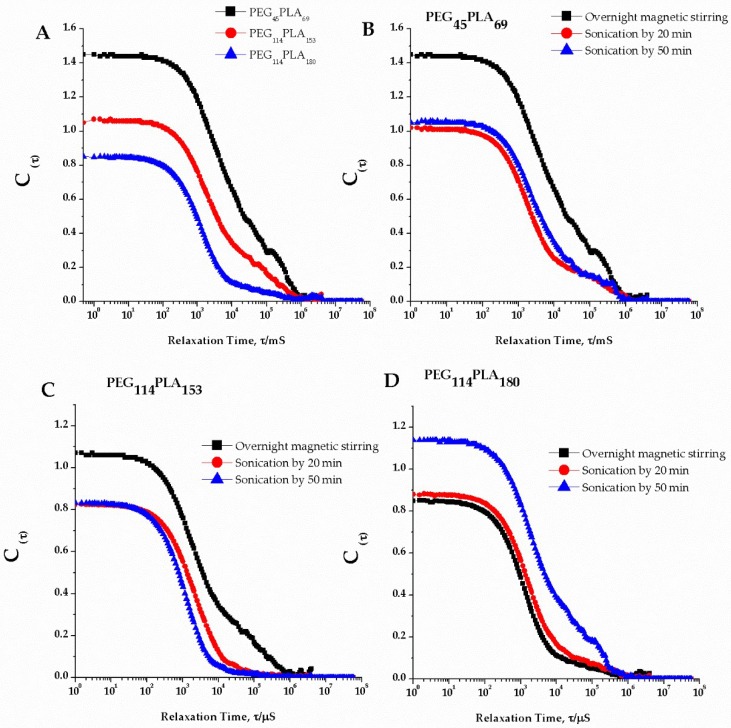
Correlation coefficients: (**A**) Decaying time for nanostructures formed from the copolymers poly (ethylene-block-lactic acid) under overnight magnetic stirring featuring the tails attributed to large particles; (**B**) Sonication effect for poly ethylene-block-lactic acid) PEG_45_PLA_69_ of MW 2000:5000 Da; (**C**) Sonication effect for poly (ethylene-block-lactic acid) of PEG_114_-PLA_153_ MW 5000:11,000 Da; (**D**) Sonication effect for poly (ethylene-block-lactic acid) of PEG_114_-PLA_180_ MW 5000:13,000 Da.

According to the ANOVA, the PDI values for the three copolymers are statistically different (*p* > 0.05) and Tukey test confirmed that PDI for PEG_45_PLA_69_ is different from the other two copolymers. The correlation function (Figure 2A) confirmed this result, showing difference in the decay times: nearly 180 µs for PEG_45_-PLA_69_ and 100 µs for PEG_114_-PLA_153_ and PEG_114_-PLA_180_. The correlation function was widely discussed by Bhattacharjee (2016) [16] and is a factor that indicates clear differences among samples. The autocorrelation decays more slowly for bigger particles due to slower relaxation of the particle dynamics.

A tail was identified in the correlation function for all copolymers emphasizing that all systems contained large particles that contributed to high PDI. Usually high PDI values in PL systems might be attributed to the presence of micelles, as reported by Bartenstein [11], but in our study we attributed these to larger structures as no sign of micelles was observed. In addition, although ANOVA indicated statically different PDI values, these values were higher than ~0.4 for the three copolymers indicating that all the samples still have a very broad size distribution (a monodisperse system would have a PDI < 0.3 [11].

These results indicated that, from the microscopic point of view, film hydration conditions were still unsatisfactory and even though higher shear of stirring improved the PDI values, other efforts would be necessary to achieve more homogenous PL preparations. 

### 3.2. Self-Assembly by Film Hydration under Magnetic Stirring and Sonication

In order to control size distribution, sonication was considered as a next step and the DLS results are presented in Table S2. Literature shows that sonication reduces aggregates size due to cavitation, which consists in the oscillation of small gas bubbles by expansion and contraction in a liquid exposed to acoustic pressure waves. The gas bubbles eventually collapse resulting in high pressures and this stress breaks up large vesicle aggregates into small vesicles [17].

The correlation coefficient (Figure 2B–D), however, was slightly different after sonication, with smoother curve fits for all sonicated preparations, except PEG_114_-PLA_180_. This could correspond to the narrower size distribution for the smoother curves. Decaying time remained approximately 180 µs for PEG_45_PLA_69_ and 100 µs for PEG_114_-PLA_153_ and PEG_114_-PLA_180_ after sonication steps, as well as tails in the correlograms. The difference in the intercepts was not considered in this study since the concentration of the samples submitted to DLS was not controlled. 

**Figure 3:**
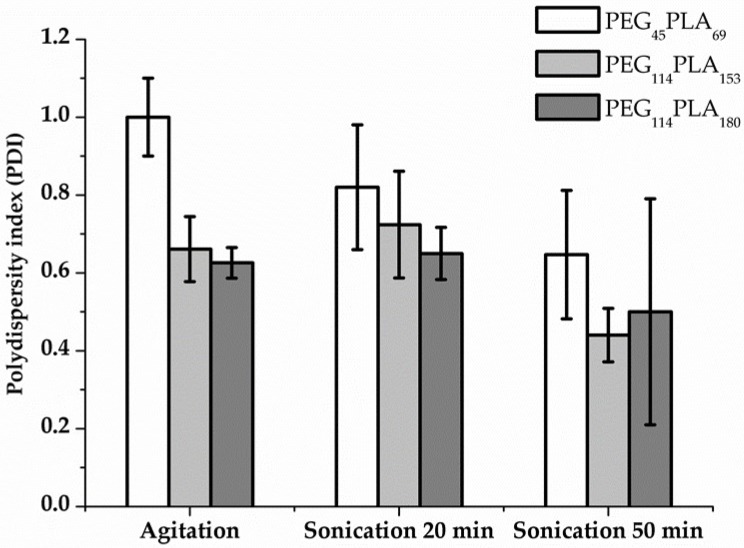
Polydispersity index (PDI) for the three copolymers poly (ethylene-block-lactic acid)-PEG-PLA under overnight magnetic stirring and different sonication times (*n* = 3).

For PEG-PLA PL, no significant differences in PDI values were observed after sonication (Figure 3) neither between the different times of sonication (20 min and 50 min).

**Figure 4:**
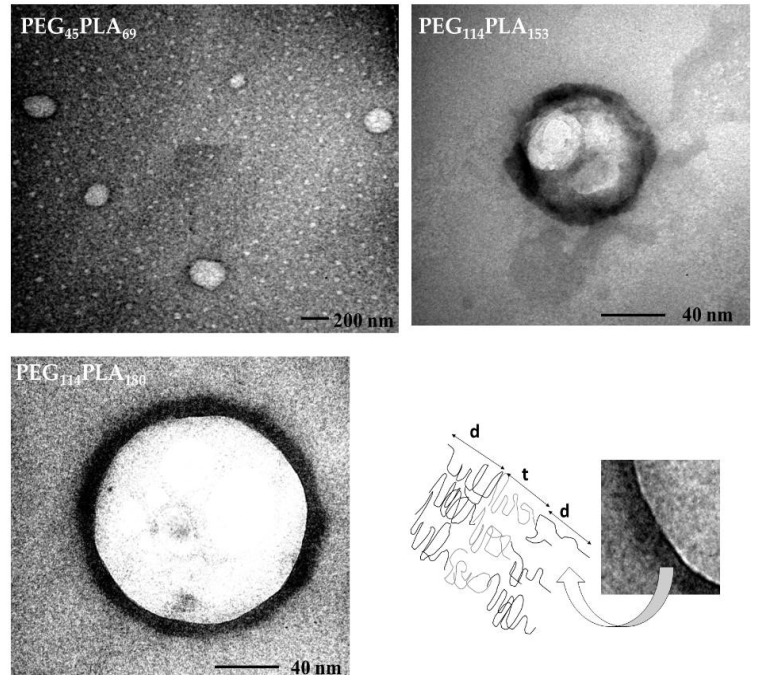
Transmission electron microscopy (TEM) images of polymersomes of poly (ethylene-block-lactic acid): PEG_45_-PLA_69_, PEG_114_-PLA_153_ and PEG_114_-PLA_180_ under overnight magnetic stirring and sonication for 50 min. The schematic representation highlights the hydrophobic membrane (t) and the hydrophilic corona of PEG (d).

The TEM images of the systems obtained under overnight magnetic stirring and sonication by 50 min are shown in Figure 4. Typical morphology of vesicular structures was confirmed and the measurements done with the Image J^®^ program indicated mean diameters about 226 nm, 94 nm and 133 nm with membranes thickness (t) of 8, 10.5 and 14 nm for PEG_45_-PLA_69_, PEG_114_-PLA_153_ and PEG_114_-PLA_180_, respectively. 

Polymersomes were found to be smaller from TEM analysis as compared to DLS, which is expected since DLS estimates hydrodynamic diameter and, consequently, the hydrophilic corona of hydrated PEG or brush thickness (d) is taken into account which leads to overestimation of the size (there are on average three water molecules per ethylene oxide unit) [18].

The TEM imaging allowed visualizing the core of the vesicles and its membrane (Figure 4, (t) -Bottom right). The preparation of TEM grids with phosphotungstic acid stain leads to negative contrast images, however the darker color of the PL membrane can be explained by the fact that phosphotungstic acid could react more strongly with the ester groups of PLA, which in this case creates positive contrast-like images for the hydrophilic corona of the PL [18]. 

**Figure 5:**
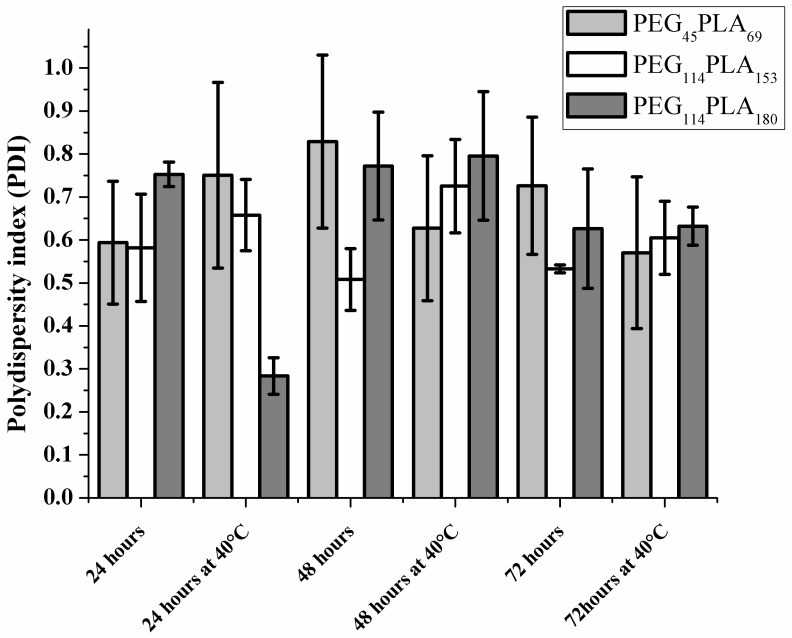
Polydispersity index for three copolymers of poly (ethylene-block-lactic acid)-PEG-PLA under magnetic stirring at different times and under heating at 40 °C (*n* = 3).

To overcome broad distribution (high PDI values), different times of magnetic stirring at 400 rpm were investigated, namely 24, 48 and 72 h of magnetic stirring (400 rpm) at room temperature or at 40 °C. Results are presented in Figure 5 and show that stirring for 24 h results in PDI values smaller than overnight (15 h) agitation. For PEG_45_-PLA_69_ and PEG_114_-PLA_153_, a decrease in PDI was observed altogether with a visual enhance in homogeneity. Although the broad size distributions and the presence of more than one size peak (Table S3–S5) was still the case, the longer stirring time facilitated hydration of the copolymer initially broken from the network of bulk film into smaller, more uniform structures. The hydrophobic block of PEG_114_-PLA_180_ has the highest molecular weight and glass-transition temperature among the three copolymers, consequently hydration of this glassy copolymer in aqueous solution even at longer times might result in the dispersion of bulk polymer film rather than in the formation of defined spherical unilamellar PL. 

**Figure 6:**
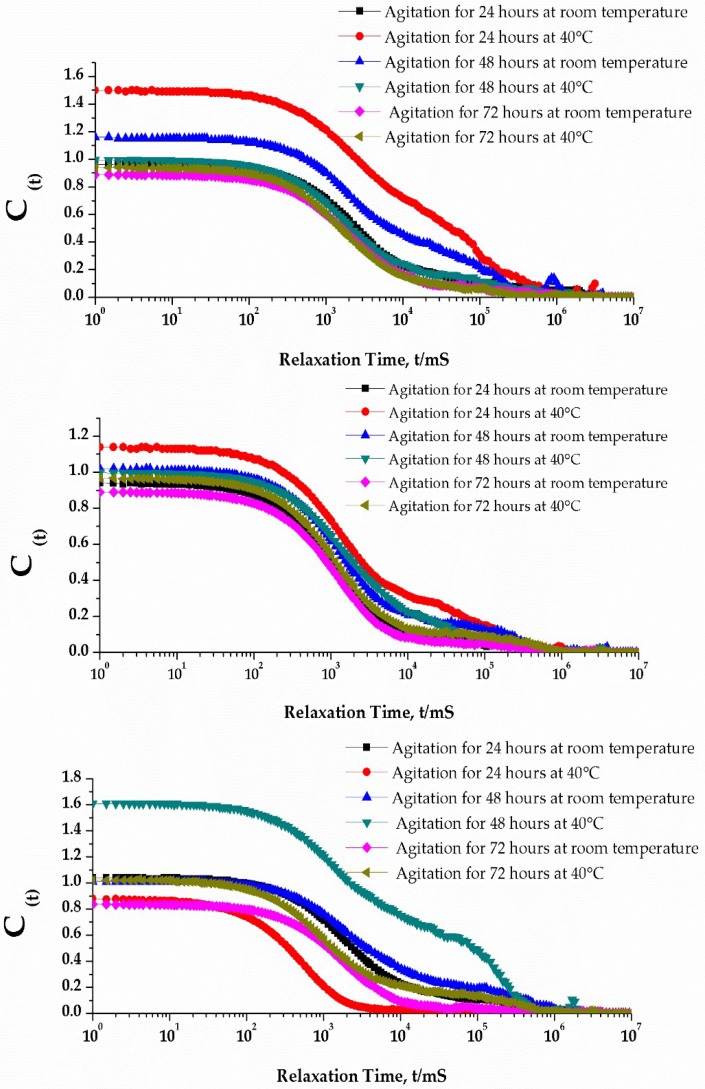
Correlation coefficients for nanostructures formed from the three copolymers of poly (ethylene-block-lactic acid): PEG_45_-PLA_69_, PEG_114_-PLA_153_ and PEG_114_-PLA_180_ under stirring at 24, 48 and 72 h with or without heating.

According to our results, stirring times higher than 24 h, did not further improve the broad size distributions and the presence of more than one size population for these systems remained. The ANOVA pointed no statistically significant difference for PDI values of PEG_45_-PLA_69_ and PEG_114_-PLA_153_ for the stirring times of 24, 48 and 72 h. For the PEG_114_-PLA_180_, a significantly lower PDI value (*p* < 0.05) was observed after 24h of stirring at 40 ^°^C, perhaps because of higher copolymer glass transition temperature. Figure 6 shows the correlation coefficients and one can see slight differences of decay time as well as improved curve smoothness for PEG_114_-PLA_180_ indicating more homogenous samples. Tables S3, S4 and S5 show the presence of peaks corresponding to higher sizes. 

We further considered the effect of temperature on self-assembly by comparing RT (20 °C) and 40 °C. Our results show that increasing the temperature did not significantly influence the vesicles self-assembly, since similar PDI values were obtained (Figure 5). We did not investigate temperatures higher than 40 °C since this might lead to protein denaturation. Moreover, PEG interacts with water by hydrogen bond and the interactions are weaker at higher temperatures due to the increased kinetic energy of water molecules. As a result, PEG moieties are dehydrated by temperature increase, favoring van der Waals interactions among PEG chains with the copolymer precipitation (hydrophobic effect).

Challenges for self-assembly of copolymers into PL by film hydration method can be understood through the studies of Battaglia and Ryan (2006) [19,20] regarding polymeric vesicle formation. Accordingly, hydration condition may lead to slow self-assembly by triggering incomplete formation of vesicles with initial finger instabilities (myelin) that are formed after water addition and copolymer swelling. These finger instabilities can grow and form vesicles owing to the copolymer diffusion in water and the water diffusion in the copolymer [19].

### 3.3. Effect of Centrifugation and Extrusion Post-Film Hydration 

**Figure 7:**
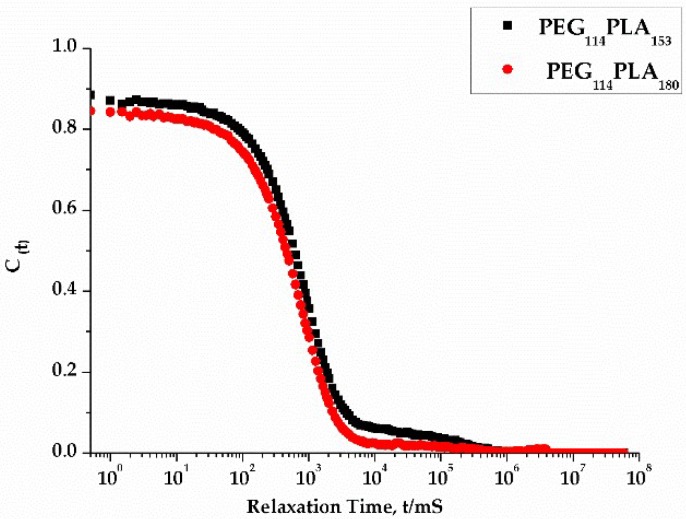
Correlation coefficients for nanostructures formed from the copolymers poly (ethylene-block-lactic acid) PEG_114_-PLA_153_ and PEG_114_-PLA_180_ after centrifugation.

Based on the results above, 24 h stirring time at room temperature was chose as the preferred condition for the following experiments. Further efforts to reduce PDI and, consequently, obtain a narrower size distribution focused on fractionation of the populations of nanostructures by centrifugation. This technique can separate PLs and bulk polymer film structures. Figure 7 shows that centrifugation resulted in PDI values of 0.363 and 0.280 for PEG_114_-PLA_153_ and PEG_114_-PLA_180_, respectively and decay times around 48 µs. Centrifugation was fast, simple and resulted in successful separation of the polydisperse aggregates/material. Unfortunately, DLS analysis could not be performed for PEG_45_-PLA_69_ because centrifugation resulted in very diluted samples due to the loss of significant material in the bulk polymeric film fraction. 

**Figure 8:**
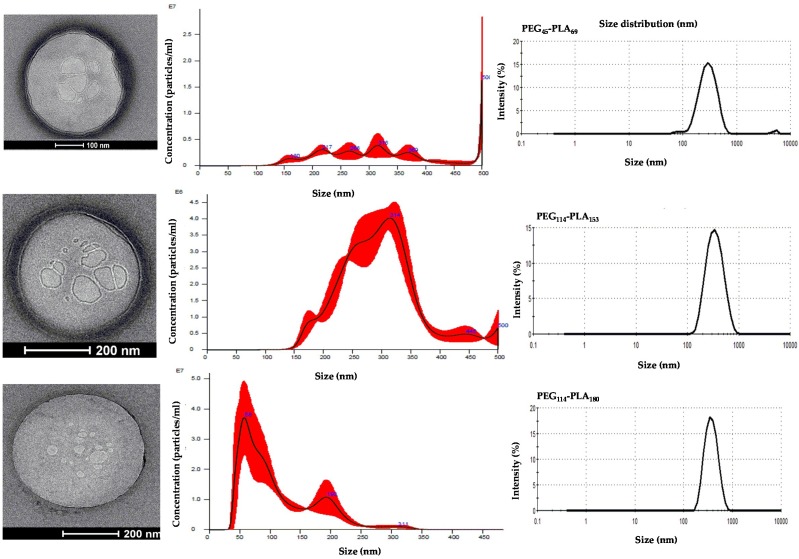
Nanoparticle Tracking Analysis, transmission electronic microscopy and dynamic light scattering by intensity of polymersomes formed from the copolymers poly (ethylene-block-lactic acid) by film hydration under magnetic stirring for 24 h at room. Samples were extruded for 31 times through 400 nm pore radii polycarbonate membrane.

Similarly, extrusion as a post-hydration method resulted in the loss of material and dilution of PL samples, confirmed by TEM images (Figure S2). To evaluate the extrusion effect, we increased the initial PEG-PLA concentration to 0.1%. Moreover, we used nanoparticle tracking analysis (NTA) to complement DLS studies (Figure S3). For PEG_114_-PLA_153_, the mean diameter values based on NTA are in agreement with the values obtained by DLS. However, significant populations of different sizes are observed by NTA for the other two copolymers. One should keep in mind that DLS is more adequate than NTA for the determination of nanostructures hydrodynamic diameter. Nonetheless, NTA allows an estimation of the concentration of PL [21] and our results clearly show that the PEG_45_-PLA_69_ system after the extrusion resulted in more diluted systems in terms of number of polymersomes due to the initial loss of polymeric material in the bulk fraction after the film hydration, what was already visually observed. The PLs prepared with higher concentration of copolymer (0.1% (*m*/*v*)) (Figure 8) resulted in narrower size distribution and PDI values of 0.345, 0.144 and 0.081 for PEG_45_-PLA_69_, PEG_114_-PLA_153_ and PEG_114_-PLA_180,_ respectively. However, for PEG_45_-PLA_69_, a tail in the distribution profile and correlogram was still observed and attributed to dust traces, which could be removed by filtration before the measurement. Here we decide not to filter the samples since it usually dilutes PL systems and may affect the vesicle shape, size or concentration (Figure S4). 

Both centrifugation and extrusion lowered the PDI values, however the mechanisms are different. The first one is a purification step with the separation of fractions by size and density [22]. In extrusion, on the other hand, larger aggregates either deform or break up, and reassemble [10]. We demonstrate that, although centrifugation and extrusion were reported as steps that generally reduce the polydispersity of PL systems, the presence of excess of bulky polymeric film leads to low concentrations of vesicles and is a limiting factor for taking this method any further. It is important to highlight that usually, for copolymers having a glassy amorphous component, extrusion should be performed above the glass transition temperature, as it was done for PEG_45_-PLA_69_ (Tg = 23 °C) and PEG_114_-PLA_153_ (Tg = 39 °C). Considering PL for protein-based drug delivery, preparation methods at temperatures higher than 40 °C were considered unsuitable due to very likely denaturation effect on the protein, therefore, extrusion was performed at 40 °C for PEG_114_PLA_180_ (Tg = 40 °C). Nonetheless our DLS results confirmed that this temperature was sufficient to avoid PL deformation in the extrusion procedure. 

**Scheme 1:**
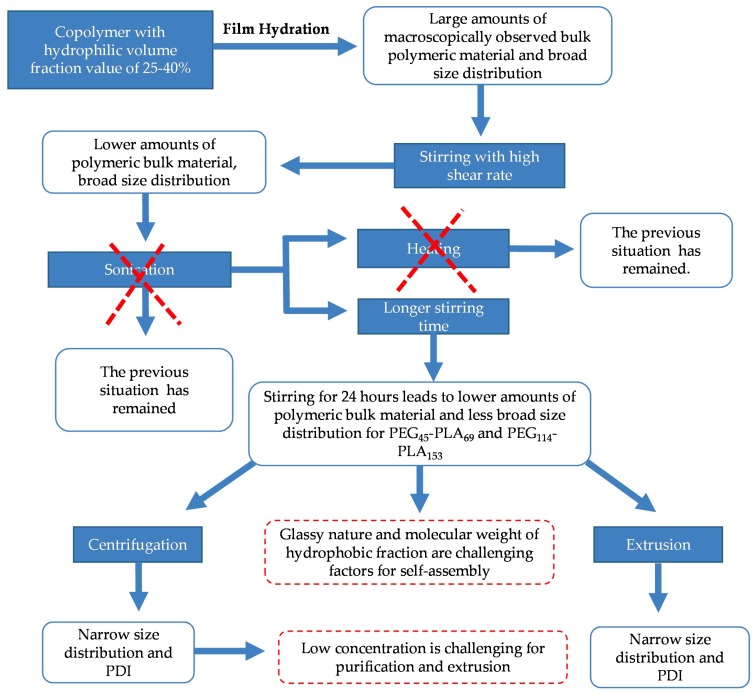
Challenges of self-assembly of poly (ethylene glycol)–poly(lactic acid) (PEG-PLA) into polymersomes.

To sum up all the conditions investigated, Scheme 1 presents the main challenges for PLs preparation from PEG-PLA copolymers self-aggregation. 

### 3.4. Protein Encapsulation into PEG-PLA Polymersomes 

**Figure 9:**
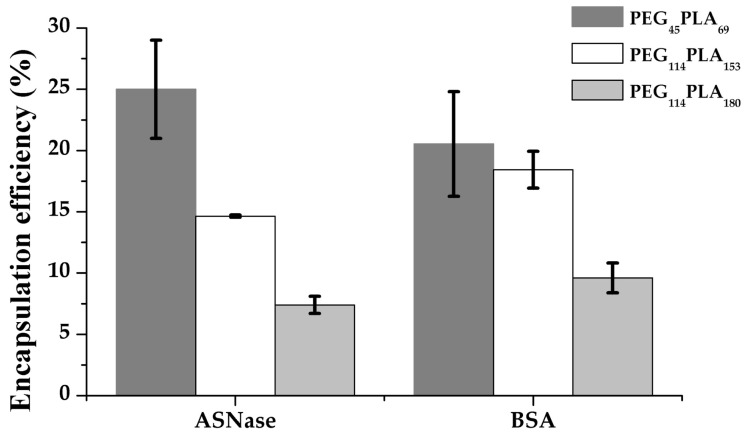
Encapsulation Efficiency values for l-asparginase (ASNase) and bovine serum albumin (BSA) in PEG-PLA polymersomes (*n* = 3).

As a proof of concept for PL formation, we encapsulated BSA and ASNase in the PEG-PLA PL (Figure 9). Our results confirm PL formation and the capacity to encapsulate globular proteins within the enclosed water volume and the encapsulation Efficiency (EE%) values presented here are in agreement with previously described in the literature for globular proteins [22]. Higher EE% values were observed for PEG_45_-PLA_69_, which corresponds with the larger vesicle size and indicates that size is directly related to the volume of the aqueous core. Additionally, one can notice that for all the PEG-PLA copolymers similar EE% values were obtained for BSA and ASNase, reinforcing that encapsulation depended mainly on the protein concentration in solution and, in this case, did not relate to protein size and/or molecular weight. Therefore, to increase EE% in PEG-PLA PL one must consider working with more concentrated protein solutions, which might present challenges due to increasing likelihood of protein aggregation.

## 4. Conclusions

In this work, we investigated the self-assembly of poly (ethylene glycol)–poly (lactic acid) (PEG-PLA) copolymers into polymersomes. Our results show that to prepare polymersomes one cannot base the preparation protocol only on the classical concepts of spontaneous self-aggregation of amphiphiles in water. When compared to other amphiphiles, block copolymers present more complex nature and other properties must be taken into account. This can include hydrophilic fraction, molecular weight or glass transition temperature of the hydrophobic block. Moreover, our work shows that the adjustment of post-preparation methods such as stirring (shear force), extrusion, sonication and centrifugation all contribute to the final characteristics of the polymersomes as well as the final yield. For instance, glass transition of the copolymer might change how difficult self-assembly is, and in some cases might result in loss of material in the form of bulk polymer. Post-film hydration techniques, such as extrusion and centrifugation, might remove bulk material and improve the homogeneity of the final preparation, but might also result in the dilution of the final system obtained. Additionally, one should consider that in spite of the self-assembly challenges, PEG-PLA copolymers of smaller hydrophilic fraction (*f*) result in higher encapsulation efficiency for hydrophilic molecules, such as proteins, which is desired for the development of therapeutic strategies. All these issues should be taken into account carefully when designing a polymersome-based system for pharmaceutical technology development. 

## Figures and Tables

**Table 1:** Molecular characteristics of the copolymers poly(ethylene glycol)–poly(lactic acid) (PEG-PLA).

Copolymer	PEG-PLA Mn ^a^	Glass Transition Temperature of Hydrophobic Block ^b^	*f_PEG_*	PDI ^c^
PEG_45_PLA_69_	PEG 2000: PLA 5000	23 °C	0.28	1.20
PEG_114_PLA_153_	PEG 5000: PLA 11,000	39 °C	0.30	1.15
PEG_114_PLA_180_	PEG 5000: PLA 13,000	40 °C	0.27	1.16

Manufacture’s data: ^a^ From 1H-NMR spectroscopy by comparing the peak area of the methoxyl protons of poly (ethylene oxide) at about a 3.6 ppm with the polylactide protons at about 5.1 and 1.55 ppm. ^b^ Determined by differential scanning calorimeter (DSC).^c^ Obtained from size exclusion chromatography (SEC).

**Table 2:** Nanostructures formed by poly (ethylene glycol)-poly(lactic acid) (PEG_45_-PLA_69_, 2000:5000 MW) through film hydration under orbital agitation at 150 RPM.

Systems	Hydrodynamic diameter by intensity (nm)	PDI
P1 2.0	836.7 (65.1%) and 213.5 (34.9%)	0.962
P1 2.20	300 (71.9%) and 1878 (28.1%)	1
P1 2.50	467.6 (94.8%) and 66.28 (5.2%)	1
P1 *ON*.0	217 (61%) and 664 (39%)	0.749
P1 *ON*.20	378.7 (69.7%) and 173.6 (30.3%)	0.673
P1 *ON*.50	443.9 (80.3%) and 146.6 (19.7%)	0.807

2: stirring for 2 h ON- overnight stirring and 20: stirring for 20 min, 50 stirring for 50 min. PDI-Polydispersity index.

## Supplementary Materials

The following are available online at http://www.mdpi.com/2079-4991/8/6/373/s1, Figure S1: Aspects of poly(ethylene glycol)–poly(lactic acid) (PEG-PLA): A) After orbital stirring at 150 RPM and B) After magnetic stirring at 400 RMP, Table S1: Dynamic Light Scattering profile and respective polydispersity index (PDI) of nanostructures formed by poly(ethylene glycol)-poly(lactic acid) (PEG-PLA) by film hydration under magnetic stirring at 400 RPM after overnight, Table S2: Dynamic Light Scattering profile and respective polydispersity index (PDI) of nanostructures formed by poly(ethylene glycol)-poly(lactic acid) (PEG-PLA) by film hydration under magnetic stirring at 400 RPM and different times of sonication, Table S3: Dynamic Light Scattering profile and respective polydispersity index (PDI) of nanostructures formed by PEG_45_-PLA_69_ (2000:5000 MW) by film hydration under magnetic stirring at 400 RPM at different times and temperatures, Table S4: Dynamic Light Scattering profile and respective polydispersity index (PDI) of nanostructures formed by PEG_114_-PLA_153_ (5000:11,000 MW) by film hydration under magnetic stirring at 400 RPM at different times and temperatures, Table S5: Dynamic Light Scattering profile and respective polydispersity index (PDI) of nanostructures formed by PEG_114_PLA_153_ (5000:11,000 MW) by film hydration under magnetic stirring at 400 RPM at different times and temperatures.Figure S2: Polymersomes from poly (ethylene glycol)–poly (lactic acid) 5000:11,000 after extrusion through 0.4 µm pores, Figure S3: Nanoparticle Tracking Analysis for particles after centrifugation: A) 0.1 % (*m*/*v*) B) at 0.03 % (*m*/*v*), Figure S4: Correlation coefficients: Decaying time for nanostructures formed from three copolymers poly (ethylene-block-lactic acid) 2000:5000 (black), 5000:11,000 (red) and 5000:13,000 (blue).
