# Supplementary material for: Challenges for the Self-Assembly of Poly(Ethylene Glycol)–Poly(Lactic Acid) (PEG-PLA) into Polymersomes: Beyond the Theoretical Paradigms
Source: Nanomaterials (Basel). 2018 May 26;8(6):373. doi: 10.3390/nano8060373 (PMC6027356; doi:10.3390/nano8060373)
Supplement: Supplementary file 1 [file nanomaterials-08-00373-s001.zip › nanomaterials-298336-SI.pdf]

nanomaterials

Supporting Information

Challenges for the self-assembly of poly (ethylene glycol)–poly (lactic acid) (PEG-PLA) into polymersomes: beyond the hydrophilic volume fraction (*f*)

Alexsandra Conceição Apolinário[1], Monika S Magoń[2,3], Adalberto Pessoa Jr.[1] and Carlota de Oliveira Rangel-Yagui[1,*]

[1] Department of Biochemical and Pharmaceutical Technology, School of Pharmaceutical Sciences, University of São Paulo, Av. Prof. Lineu Prestes, 580 - Bl.16, CEP: 05508-000, Brazil; acapolinario@gmail.com

[2] Department of Chemistry, University College London, Christopher Ingold Building, 20 Gordon Street WC1H 0AJ, London, United Kingdom; m.magon.12@ucl.ac.uk

[3] Current address: BSRC Complex, School of Biology, University of St Andrews, St Andrews KY16 9ST, United Kingdom

* Correspondence: corangel@usp.br; Tel.: + 55 11 30912381

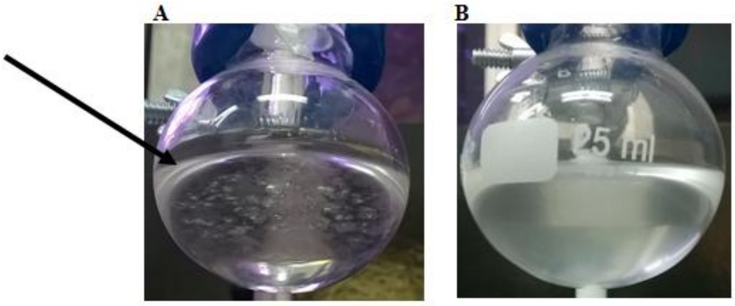

Figure S1. Aspects of poly(ethylene glycol)–poly(lactic acid) (PEG-PLA) system formed by film hydration by A) orbital stirring at 150 RPM and B) magnetic stirring at 400 RMP.

Table S1. Dynamic Light Scattering profile and respective polydispersity index (PDI) of nanostructures formed by poly(ethylene glycol)-poly(lactic acid) (PEG-PLA) by film hydration under magnetic stirring at 400 RPM after overnight.

Copolymer	Scattering Intensity (nm)	Scattering by Number (nm)	PDI
**$PEG_{45}PLA_{69}$**	466 (100%)	416.1 (100%)	1
**$PEG_{114}PLA_{153}$**	558.4 (85.1 %) and 155.4 (14.9%)	200.9 (100 %)	0.771
**$PEG_{114}PLA_{180}$**	439.9 (96.9%) and 5331 (3.1%)	185.7 (100%)	0.609

Table S2. Dynamic Light Scattering profile and respective polydispersity index (PDI) of nanostructures formed by poly(ethylene glycol)-poly(lactic acid) (PEG-PLA) by film hydration under magnetic stirring at 400 RPM and different times of sonication.

Copolymer	Sonication by 20 min		Sonication by 50 min	
	Scattering Intensity (nm)	Scattering by Number (nm)	Scattering Intensity (nm)	Scattering by Number (nm)
**PEG_{45}-PLA_{69} (2000:5000 MW)**	600.6 (46 %) 380(45.2%) and 186 (8.8%)	165(50.6%) and 400 (49.4%)	376 (58 %), 1050 (30%) and 4379 (12%)	341 (94 %) and 1024 (6%)
**$PEG_{114}PLA_{153}$ (5000:11000 MW)**	1081 (62.8%), 276.5 (28.7 %) and 4788 (8,5 %)	952.1 (7.8%), 226.5 (92.1%) and 4601 (0.1%)	659.4 (57%), 245.4 (35.3%) and 5059 (7.8%)	232 (99%) and 5005 (0.1%)
**$PEG_{114}PLA_{180}$ (5000:13000 MW)**	446(99 %) and 5560 (1%)	218 (100%)	454 (95.4%), 5298 (2.6%) and 86 (2%)	204 (70%) and 75 (30%)

Table S3. Dynamic Light Scattering profile and respective polydispersity index (PDI) of nanostructures formed by PEG_{45}-PLA_{69} (2000:5000 MW) by film hydration under magnetic stirring at 400 RPM at different times and temperatures.

PEG_{45}-PLA_{69} (2000:5000 MW)	Scattering Intensity	Scattering by Number
**Agitation for 24 hours at room temperature**	572 (100%)	491.6 (100%)
**Agitation for 24 hours at 40°C**	390.2 (96%), 122.6 (3%) and 5560 (1%)	106.6 (31%) and 326 (69%)
**Agitation for 48 hours at room temperature**	467 (97%) and 155 (3%)	415 (64%) and 164 (36%)
**Agitation for 48 hours at 40°C**	460 (95%), 4919(4%) and 108 (1%)	392 (70%) and 90 (30%)
**Agitation for 72 hours at room temperature**	752 (56%) and 335 (44%)	324 (100%)
**Agitation for 72 hours at 40°C**	758 (62%), 270 (37%) and 5296 (1%)	286 (100%)

Table S4. Dynamic Light Scattering profile and respective polydispersity index (PDI) of nanostructures formed by $PEG_{114}PLA_{153}$ (5000:11000 MW) by film hydration under magnetic stirring at 400 RPM at different times and temperatures.

$PEG_{114}PLA_{153}$ (5000:11000 MW)	Intensity	Number
**Agitation for 24 hours at room temperature**	362 (100%)	110 (51%) and 283 (49%)
**Agitation for 24 hours at 40°C**	349 (93%) and 109 (7%)	280 (37%) and 93 (63%)
**Agitation for 48 hours at room temperature**	357.5 (100 %)	140 (19%) and 294 (81%)
**Agitation for 48 hours at 40°C**	567 (73%) and 176 (27%)	520 (6%) and 138 (94%)
**Agitation for 48 hours and sonication for 50 min at 40°C**	309 (92%) and 4224 (8%)	66.44 (30%) and 142 (70%)
**Agitation for 72 hours at room temperature**	401 (94%) and 5321 (6%)	136 (100%)
**Agitation for 72 hours at 40°C**	574 (52%), 234 (47%) and 5560(1%)	192 (100%)

Table S5. Dynamic Light Scattering profile and respective polydispersity index (PDI) of nanostructures formed by $PEG_{114}PLA_{153}$ (5000:11000 MW) by film hydration under magnetic stirring at 400 RPM at different times and temperatures.

$PEG_{114}PLA_{180}$ (5000:13000 MW)	Intensity	Number
**Agitation for 24 hours at room temperature**	745 (59%).276 (40%) and 5526 (1%)	246 (86%) and 670 (14%)
**Agitation for 24 hours at 40°C**	200.6 (100 %) and 5448 (1%)	128 (80%) and 53 (20%)
**Agitation for 48 hours at room temperature**	596 (98) and 167(2%)	376(84 %) and 160 (16%)
**Agitation for 48 hours at 40°C**	229 (83%) and 623.4 (17%)	115 (25%) and 218 (75 %)
**Agitation for 72 hours at room temperature**	450 (60%), 216 (36%) and 5156 (4%)	177 (100%)
**Agitation for 72 hours at 40°C**	325 (78.5%) and 129.5 (21%)	119 (100%)

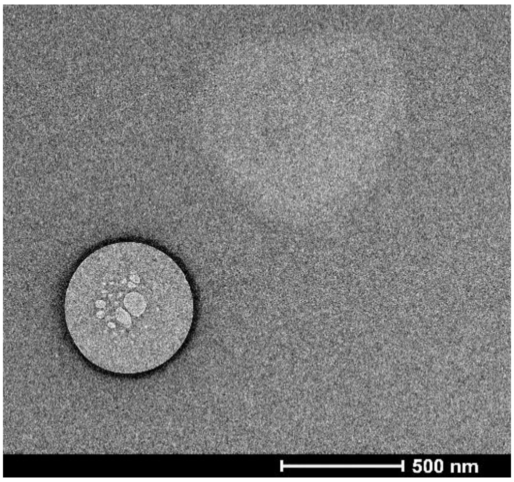

Figure S2. Polymersomes from poly(ethylene glycol)-poly(lactic acid) $PEG_{114}PLA_{153}$ after extrusion through 0.4 μm pores.

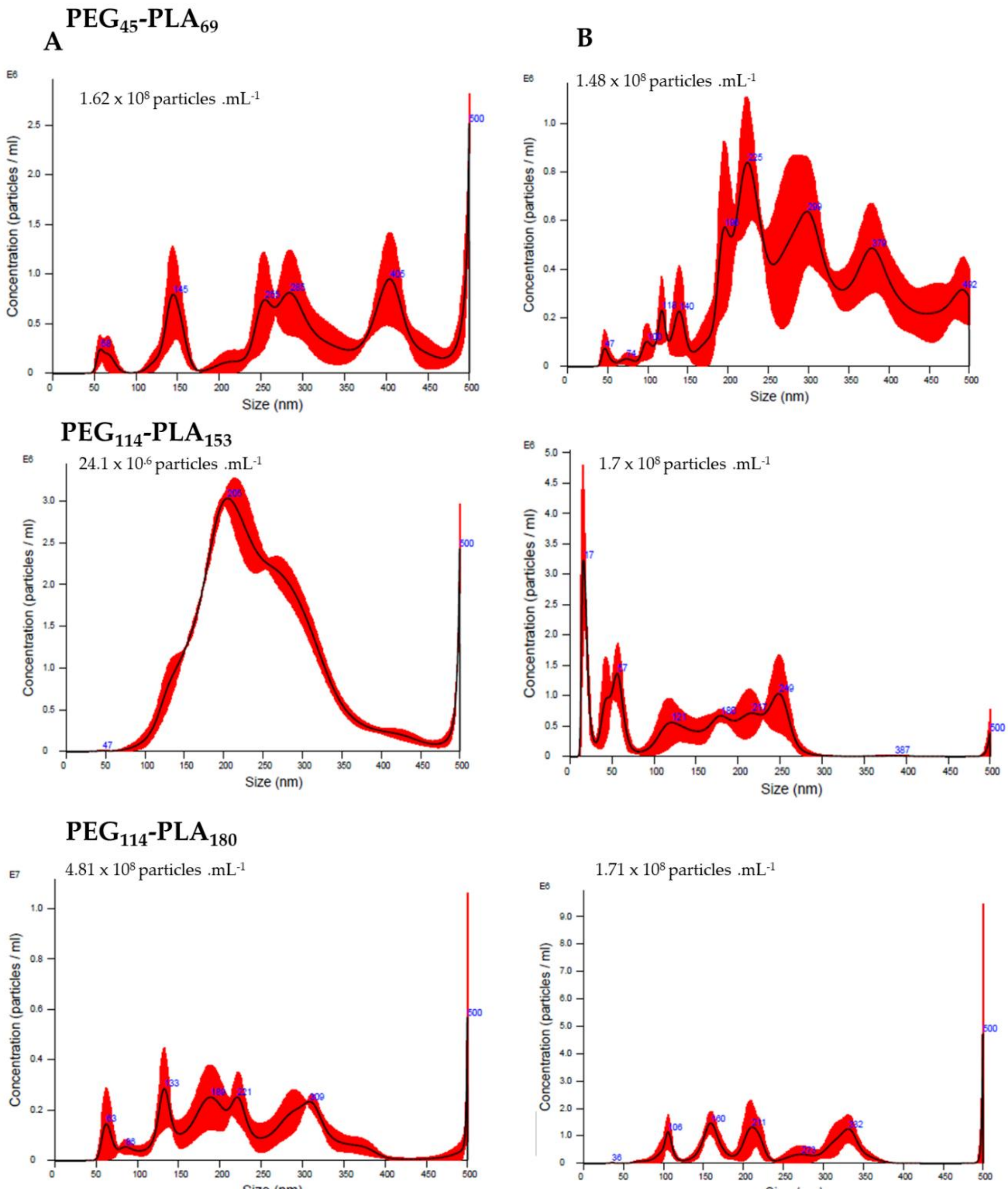

Figure S3. Nanoparticle Tracking Analysis of poly(ethylene glycol)-poly(lactic acid) (PEG-PLA) nanostructures after centrifugation: A) 0.1 % (m/v) B) At 0.03 % (m/v).

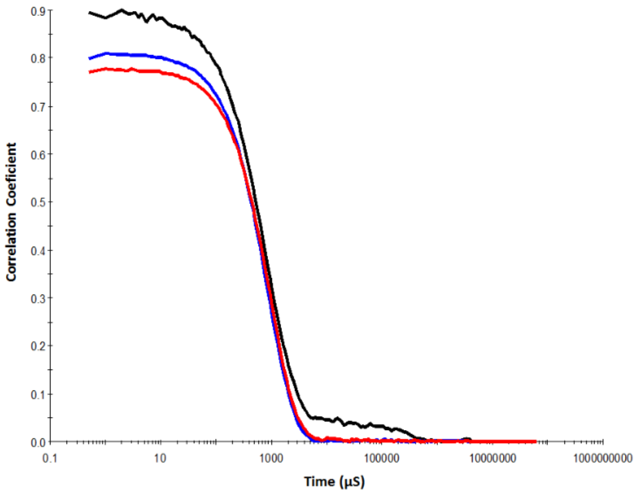

Figure S4. Correlation coeficients: Decaying time for nanostructures formed from PEG_{45}-PLA_{69} (black), $PEG_{114}PLA_{153}$ (red) and $PEG_{114}PLA_{153}$ (blue).
